# Scanning electron microscopy of hyphal ectobiont bacteria within mycelial extracellular matrices

**DOI:** 10.1016/j.bpr.2025.100233

**Published:** 2025-09-11

**Authors:** Davin Browner, Andrew Adamatzky

**Affiliations:** 1 Unconventional Computing Laboratory, UWE, Bristol, United Kingdom

## Abstract

Fungi and bacteria are found living in a wide variety of environments, and their interactions are important in many processes including soil health, human and animal physiology, and in biotechnological applications. Here, we investigate a single morphological feature of cocultures of planktonic bacterial growth within biofilm-forming liquid cultures of mycelium, namely, the attachment of bacterial ectobionts of species *Bacillus subtilis* to fungal hyphae of species *Hericium erinaceus*. The bacteria-in-mycelial-biofilm method was developed and utilized to allow for attachment of bacteria to hyphae via containment within exopolymeric substances (EPS) and the overall extracellular matrix of the mycelium. A graded dehydration protocol was used to selectively remove extraneous biofilm components and reveal intact bacteria and surface-interfacing features. The dehydration methods allowed for identification of specific interactions and differentiated these cultures from trivial stochastic mixing of bacteria and mycelium in liquid media. Attachment structures appear to be produced primarily by the mycelium and enveloped the bacterial ectobiont. Nanoscale surface-interfacing EPS constituents were preserved, providing a biophysical basis for a range of contact-dependent modulating properties of the bacteria on this fungal host. The mean biofilm area across triplicates was $3.90\;\mu m^{2}\pm 0.72\;\mu m^{2}$, and the mean percentage coverage was 18.33%±5.52%. The bacterial biofilm components could not be ruled out as co-contributing to formation of attachment structures due to the structures being present connecting individual bacteria as well as to hyphae.

## Why it matters

Fungal-bacterial interactions, formed through the physical attachment of bacterial ectobionts on fungal hyphae surfaces, play a crucial role in various ecological settings, as well as in clinical cases of fungal diseases and industrial applications. However, distinguishing ectobionts from stochastic mixing or other trivial interactions can be challenging. To address this issue, we developed a method to assess the attachment of bacteria to biofilm-forming mycelial hyphae. We injected bacteria into a growing biofilm of liquid-cultured mycelium, and a chemical dehydration protocol was employed to selectively remove the outer layers of the biofilm, preserving the structures related to bacterial attachment, the bacteria and mycelium. This protocol confirmed the presence of ectobiont bacteria and related nanoscale surface-interfacing structures across triplicate cultures.

## Introduction

Bacterial microorganisms occur in almost all ecological settings, and their ubiquitous presence has led to antagonistic and mutualistic relationships with fungi. These interactions are important components in a large number of environmental processes (1,2). Co-communities have been described to exist in virtually all ecosystems and across a wide diversity of fungal and bacterial families. In artificial settings, fungal and bacterial communities of microorganisms have been used in food production and agriculture (3), pharmacological (4), bioremediation (5), and biotechnological applications (6).

When directly observed, the bacterial and fungal interactions appear to intrinsically modulate the behavior of either or both of the microorganisms. However, the integration of fungal and bacterial components is complicated by different levels and specificity of interactions (7). For example, stochastic mixing of bacterial components in liquid cultures of fungi is unlikely to be representative of a causal relationship (7). In contrast, co-occurrence can lead to integrated biophysical and metabolic interactions. As a result, interactions can be simple, specific, or trivial (7). Both intracellular (8) and extracellular (9) relationships have been observed. In the extracellular environment, interactions exist within a spectrum of activity relating to a variety of processes including growth, reproduction, transport, movement, nutrition, stress resistance, and pathogenicity (7). These processes impact the microorganisms at different levels and with ranging specificity resulting from the combined physical associations (biofilm and free cells), the molecular dialogue between the organisms (direct or indirect), and the environmental conditions and/or host activity (7). Ectobiont or hyphal surface-interfacing bacteria are of particular interest in extracellular studies (10).

Fungal mycelial exopolymeric substances (EPS) play a critical role in modulating bacterial biofilm architecture and antimicrobial resistance in the extracellular environment. For instance, fungal EPSs such as galactosaminogalactan can integrate into bacterial biofilms, enhancing bacterial adherence, as demonstrated in *Aspergillus fumigatus* and *Pseudomonas aeruginosa* cocultures (11). The molecular mechanisms underlying fungal-bacterial biofilm interactions involve complex biochemical signaling and physical interactions, including quorum sensing and EPS-mediated adhesion. Mechanisms of bacterial adherence to fungal structures have been observed, highlighting the role of biofilm matrix components and specific adhesins (12,13). Studies have identified specific bacterial and fungal surface proteins or polysaccharides mediating adhesion, such as fungal galactosaminogalactan, bacterial cellulose, chitin-binding proteins, and adhesins like Als3 and SspB (11,14,15). Other research highlighted the critical role of fungal cell wall components (mannoproteins and glycans) and bacterial adhesins or biofilm matrix components in establishing physical attachment (13,16,17). Bacterial secretion of molecules (e.g., diketopiperazines, gingipains) that modulated adhesion and biofilm formation was reported, indicating complex molecular cross talk (18,19). Atomic force microscopy and surface thermodynamics were used to quantify adhesion forces, revealing strong bacterial-fungal adhesion forces in the range of 4–5 nN, dependent on specific adhesins or surface proteins (14,20,21). Thermodynamic analyses showed that favorable acid-base and van der Waals interactions facilitate close bacterial approach to fungal hyphae, modulated by fungal morphology and bacterial quorum sensing molecules (20). A number of studies inferred physical forces indirectly through biofilm adherence assays or microscopy without direct force measurements (11,13).

Bacterial surface attachment has been found to alter biofilm architecture, increasing biomass, promoting multilayer biofilm formation and creating complex spatial organization (22). Fungal exopolysaccharides and extracellular DNA contribute to biofilm matrix integrity and support bacterial colonization (23). Some studies reported antagonistic effects where bacterial attachment inhibits fungal biofilm development or causes morphological alterations (24). Attachment efficiency and mechanisms vary widely across bacterial and fungal taxa, environmental contexts, and fungal morphologies, with hyphal form often critical for adhesion (25,26). Soil, marine, clinical, and plant-associated environments show distinct bacterial-fungal interaction patterns influenced by microbial community composition and environmental factors (27,28). Some bacteria preferentially attach to vital versus nonvital hyphae, and fungal species identity can modulate bacterial colonization (26). The use of diverse and complementary methodologies, including atomic force microscopy, confocal and electron microscopy, genetic mutants, and transcriptomics, has enriched understanding of bacterial-fungal physical interactions (20,29,30). Microfluidic and time-lapse imaging techniques have enabled dynamic studies of coculture development at multiple length scales (29,31,32). However, methodological limitations include challenges in studying thick, three-dimensional hyphal networks where EPS has formed, complicating imaging and quantification of biofilms and imaging of nanometer-sized attachment structures without the use of fluorescent markers (33). As a result, there is a lack of characterization of biofilm matrix composition and matrix integration in cocultures. The molecular composition and spatial organization of mixed bacterial-fungal biofilm matrices remain incompletely defined, especially regarding the integration of fungal and bacterial exopolysaccharides and physical attachment mechanisms of bacteria.

Development of protocols for assessing the physical attachment of bacteria to fungal hosts would aid investigation of the modulatory capacity of putative hyphae-attached epibionts on fungal cytoskeletal dynamics (34) and electrophysiology (35). Bacteria have previously been found to have profound effects on the electrophysiology of animal hosts (36,37,38,39). Their impact on fungal electrophysiology via attachment-dependent structures is therefore an important aspect of investigations into fungal electrical signaling and electrophysiology (35,40). Pore formation in lipid membranes by bacteria (41) and induction of cell wall stress in hyphae by bacterial ectobionts (10) are likely to have electrophysiological effects in the fungal host. Physical interactions including bacterial adhesion to fungal hyphae and biofilms are therefore critical to understanding potential effects on fungal electrophysiology and related physiology. Bacterial surface proteins and fungal adhesins may mediate selective attachment, influencing ion channel function and membrane properties (42). Physical contacts can trigger transcriptional changes and localized electrophysiological responses in fungi, highlighting the experimental importance of contact-dependent modulation (43).

In this paper, we outline methods for determining the presence of putative contact-dependent modulation structures in cocultures of bacteria grown inside mycelial biofilms in the form of overnight cultures of bacteria *Bacillus subtilis* and Basidiomycete fungus *Hericium erinaceus*. A dehydration gradient protocol was used to selectively remove the outer layers of the biofilm and expose attachment surfaces. The bacterial and mycelial structures were also preserved, enabling measurement of morphology not possible using other hydration-preserving scanning electron microscopy (SEM) approaches such as environmental scanning microscopy (ESEM). The imaged structures repeatedly show bacterial attachment to the hyphal surface via EPSacross triplicates. This confirmed the status of the bacteria as an ectobiont of this fungus and provides a biophysical basis for contact-dependent modulation of physiology of the host. Attachment structures appear to result from the hyphal surface as a result of production of EPS of a fungal origin as a result of the static submerged liquid culturing methods.

## Materials and methods

### Static submerged liquid cultures and cocultures

Mycelial cultures of *H. erinaceus* were sampled from stocks previously identified as showing biofilm-forming capacity in liquid media. This capacity was established via growth from stocks of agar plate samples in malt liquid culture media (1.5%). Aeration was limited to a single syringe filter (13 mm diameter and 0.22 *μ*m filter size). No stirring or aeration occurred, and the static submerged liquid fermentation methods resulted in the formation of biofilms in the liquid culture. Using these stocks, fungal biofilms for SEM were grown in submerged cultures within glass vials sealed with butyl rubber stoppers and incubated at 25°C for 24 hours. Then the liquid cultures were grown at room temperature ($20^{\circ }C$) for 4 days in 20-mL vials without agitation. Lack of supplementation simplified the resulting SEM analysis due to removal of impurities and trace contents during dehydration. The vials were closed with self-healing butyl rubber stoppers, which functioned as a sterile method to initiate growth via injection of liquid cultures and restricted oxygen supply to increase fermentation and EPS formation. Mycelial growth occurred in an enclosed growth tent and without exposure to light. Temperature fluctuations were avoided by use of a temperature-controlled lab set to $20^{\circ }C$. Bacterial species were grown overnight in an incubator at $35^{\circ }C$ from stocks of *B. subtilis* (LZB 025). The resulting bacterial cultures were then transferred to the vial containing the mycelial liquid cultures, and the vial was tilted and left at room temperature in a dark environment for 12 hours. Solutions of 100 *μ*L of bacterial media were transferred into each of the triplicate samples. After this resting period the coculture samples underwent further processing for SEM imaging as detailed below.

### Graded dehydration protocol

Using sterile techniques and a dissecting microscope, the cocultures were transferred from the growth vial to an empty 20-mL glass vial. The samples were fixed in 4% glutaraldehyde in PBS for 1 hour at room temperature ($20^{\circ }C$) and then rinsed three times with PBS and stored until required. Fixed cocultures were dehydrated in a graded ethanol series. Ethanol was replaced by hexamethyldisilazane in 5-minute steps in the following ratios: 1:2, 1:1, and 2:1. This was followed by two rinses in 100% hexamethyldisilazane. The cocultures were left to dry overnight on filter paper in a sealed Petri dish and mounted on SEM stubs. They were sputter-coated with gold immediately before imaging. They were then imaged using the scanning electron microscope. More than two dehydration steps were found to dehydrate the samples, resulting in complete collapse of the hyphae. The dehydration and processing steps were found to successfully remove extraneous features and were repeated for triplicates. The method allows for removal of the outer layers of biofilm and reveals the surface-interfacing structures of the bacterial ectobionts with minimal damage to target structures.

### Scanning electron microscopy preparation, parameters, and image processing

The FEI Quanta 650 FEG scanning electron microscope (FEI Company, U.S.) was used to image the samples and produce micrographs. Image processing and analysis was performed using ImageJ (44). The scale was set using the metadata from the scanning electron microscope and each sample. Measurements were then performed taking into account the 3D structure of samples. Occluded, deformed, or partially visible bacteria and hyphae were not included in measurements.

## Results

**Figure 1:**
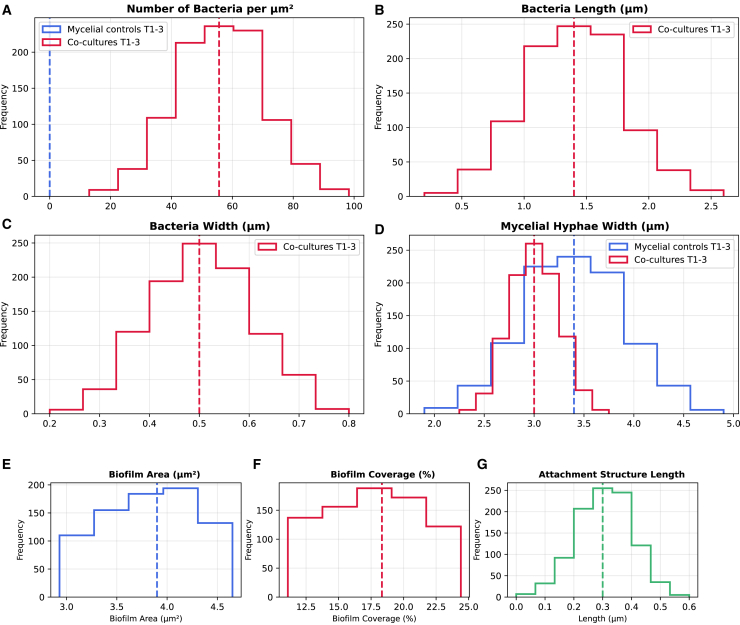
Morphometry of mycelial controls and co-cultures. (*A*) Bacteria per $\mu m^{2}$. (*B*) Bacteria length (*μ*m). (*C*) Bacteria width (*μ*m). (*D*) Hyphae width (*μ*m). (*E*) Cutoff layer remaining biofilm area $\mu m^{2}$. (*F*) Cutoff layer remaining biofilm coverage (%). (*G*) Bacterial attachment structure length.

The mean length of cocultured bacteria in the triplicate samples was 1.4 *μ*m ± 0.4 *μ*m. The smallest bacterium size was 0.53 *μ*m, and the largest observed was 2.6 *μ*m. The mean width of bacteria was 0.5μm±0.1μm. The minimum width of bacteria was 0.25μm, and the largest was 0.85μm. Bacteria with longer lengths were likely to be the result of binary fission processes that were ubiquitous in the coculture samples and indicate active physiological processes occurring before fixation. The mean width of hyphae in the cocultures across triplicates was 3μm±0.5μm compared with a mean width of hyphae in the monocultures of 3.4μm±0.4μm. The largest hyphal width observed for the monocultures was 4.5 μm, and the smallest width was 2.5 μm. For the cocultures, the maximum width of hyphae observed was 4.2 μm and smallest 2.3 μm. These measurements are outlined in in Fig. 1 *A*–*D* for mycelial monoculture controls and cocultures. We note that some effects of dehydration were not ideal and impacted the morphology of hyphae, leading to hyphae width estimations on the lower end of typical widths of 1 *μ*m–10 *μ*m. The procedure seems to have had limited effect on the bacterial cohabitants, with only sporadic instances such as burst bacteria apparent in the sample images. The dehydration gradient could be augmented to improve retention of the hyphae structures. However, the structural characteristics of the surface-interfacing bacteria were preserved sufficiently for measurements.

The morphological features of the retained biofilm area in the dehydrated cocultures are detailed for each of the triplicates in Fig. 1 *E* and *F*. The Sato ridge detection algorithm was used to detect ridge-like structures in the SEM images and to identify retained EPS constituents after dehydration. It is based on analysis of the eigenvalues of the Hessian matrix of the image. The algorithm enhances ridge-like structures while suppressing noise and other structures. The remaining biofilm components appeared as ridge-like structures contrasting hyphae and bacteria.

Given an image *I*, the Hessian matrix *H* at each pixel was defined as follows:

$$H=\left[\begin{array}{cc} \frac{\partial^{2}I}{\partial x^{2}} & \frac{\partial^{2}I}{\partial x\partial y}\\ \frac{\partial^{2}I}{\partial y\partial x} & \frac{\partial^{2}I}{\partial y^{2}} \end{array}\right]$$

The eigenvalues $\lambda_{1}$ and $\lambda_{2}$ of the Hessian matrix were computed. For ridge detection, the following conditions were used:

$$\text{if}\;\lambda_{1}\approx 0\;\text{and}\;\lambda_{2}\ll 0,\text{then}\;a\;\text{ridge}\;\text{was}\;\text{detected.}$$

The Sato filter response *R* was given by:

$$R=\left\{ \begin{array}{cc} \left|\lambda_{2}\right| & \text{if}\;\lambda_{1}\approx 0\;\text{and}\;\lambda_{2}\ll 0\\ 0 & \text{otherwise} \end{array}\right.$$

The biofilm area was calculated using custom Python code primarily utilizing the scikit-image library (also known as skimage) (45). The code performed image analysis on the data set of 1-*μ*m scale bar micrographs to calculate the area and coverage of biofilm in the images. The images were processed to convert them to grayscale, thresholded to identify biofilm components, and then analyzed to compute the area and coverage percentage.

Let *I* be an image with dimensions M×N. The scale for each image was defined as:

$$\text{scale}=\frac{1}{\text{pixels}\_\text{per}\_\text{micron}\left[image\_name\right],}$$

where pixels_per_micron was a dictionary containing the number of pixels per micron for each image.

The area of vessels $A_{v}$ in microns squared was calculated as:

$$A_{v}={\text{vessel}}_{\text{pixels}}\times {\left(\text{scale}\right)}^{2}\text{,}$$

where vessel_pixels was the number of pixels identified as vessels.

The total area $A_{t}$ in microns squared was:

$$A_{t}=M\times N\times {\left(\text{scale}\right)}^{2}$$

The percentage of vessel coverage $P_{v}$ was:

$$P_{v}=\left(\frac{\text{vessel}\_\text{pixels}}{M\times N}\right)\times 100$$

**Figure 2:**
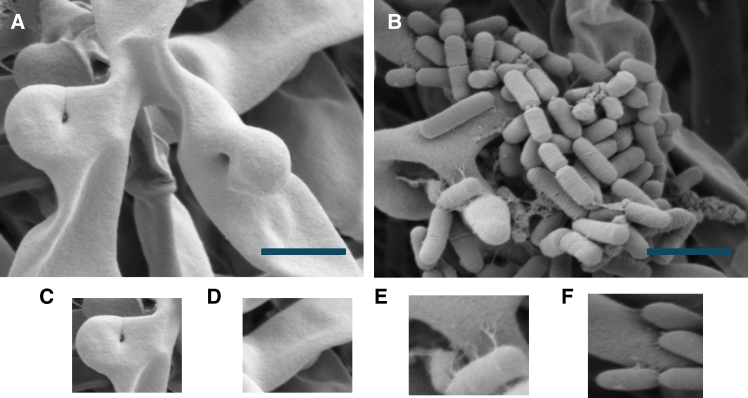
SEM micrographs. (*A*) Mycelial monocultures (5-*μ*m scale bar, magnification of ×9652, Hv of 2.00 kV, internal pressure of $2.36\times 10^{-6}\;\text{Torr,}$ and HFW of 21.6 *μ*m). (*B*) Cocultures (5-*μ*m scale bar, magnification of ×8052, Hv of 2.00 kV, internal pressure of 4.93 × 10^−6^ Torr, and HFW of 25.7 *μ*m). (*C*) Detail of hyphal branching in mycelial monoculture samples. (*D*) Detail of hyphal surface in mycelial monoculture samples. (*E*) Detail of the attachment of the bacteria to hyphae via biofilm structures. (*F*) Detail of the attachment of the bacteria to hyphae via biofilm structures.

**Figure 3:**
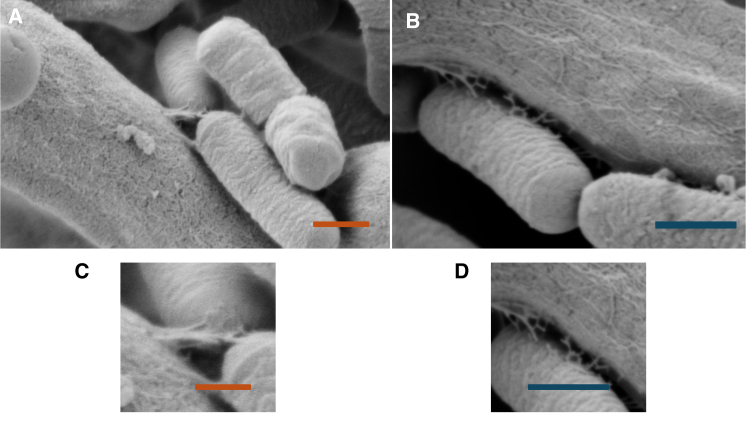
SEM micrographs of nanoscale surface-interfacing features of the bacterial ectobionts. (*A*) Cocultures (500-nm scale bar, magnification of ×51368, Hv of 2.30 kV, internal pressure of 3.44 × 10^−6^ Torr, and HFW of 4.03 *μ*m). (*B*) Cocultures (500-nm scale bar, magnification of ×32428, Hv of 2.30 kV, internal pressure of 3.21 × 10^−6^ Torr, and HFW of 6.39 *μ*m). (*C*) Zoomed detail of region of interest in (*A*) showing attachment structures. (*D*) Zoomed detail of region of interest in (*B*) showing attachment structures.

**Figure 4:**
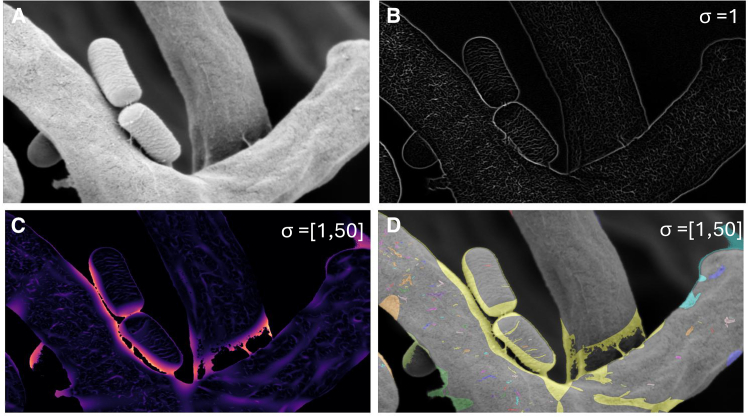
Computational analysis of co-culture micrographs. (*A*) Original micrograph. (*B*) Sato filter applied (*σ* = 1). (*C*) Identification of extracellular components distinct from the mycelia and bacteria using the Sato filter (*σ* = [1,50]). (*D*) Segmentation of biofilm components from fungal and bacterial structures based on the Sato filtering.

The mean biofilm area for triplicates was $3.90\;\mu m^{2}\pm 0.72\;\mu m^{2}\text{,}$ and the mean percentage coverage was 18.33%±5.52%. Attachment structures were measured in higher magnification images (1-*μ*m scale bar). The mean length of attachment structures to hyphae was 0.3μm±0.1μm (see Fig. 1 *G* for a detailed outline). SEM micrographs of the fungal monocultures and cocultures are shown in Fig. 2 *A*–*F*, including absence of bacteria in the fungal monocultures and attachment structures visible in the cocultures. SEM micrographs of nanoscale surface-interfacing features of the bacterial ectobionts are outlined in Fig. 3 *A*–*D*. The image processing methods used to quantify the remaining biofilm and EPS components following the dehydration protocol are shown in in Fig. 4 *A*–*D*.

## Discussion

In this paper, injection of planktonic *B. subtilis* bacteria into the extracellular matrix (ECM) of growing liquid cultures of *H. erinaceus* results in enveloping some fraction of bacteria in the ECM and attachment to hyphae. The fungal origin of the ECM may be assumed due to experimental parameters including submerged liquid culture growth methods, use of EPS-forming cultures, and confirmation of formation of EPS materials in *H. erinaceus* liquid cultures before injection. The general tendency for wild-type mycelium to form EPS in liquid cultures should also be noted for cases where sealed growth chambers are used as in this study. However, the presence of connective structures between bacteria as well as connecting bacteria to hyphae suggests that coproduction of EPS materials may have occurred.

Physical contact between bacterial and fungal cells, mediated by adhesins, surface proteins, and polar attachment, constitutes an important facet of coculture interactions in mycelial biofilms. Evidence indicates that such surface-interfacing contacts can trigger fungal secondary metabolism, providing a mechanistic basis for electrophysiological modulation beyond diffusible signals (42). This is exemplified by studies showing that physical contact induces fungal gene expression changes and secondary metabolite production, which may affect ion channel function and membrane potential (42). Microfluidic and coculture systems have begun to elucidate the spatial and temporal dynamics of these contacts, but comprehensive models connecting physical interaction to electrophysiological outcomes have not been established.

Theoretical models of microbial electrical signaling are extended by evidence of synchronized membrane potential dynamics and ion-channel-mediated electrical oscillations in bacterial biofilms, suggesting that similar electrical phenomena may underpin bacterial-fungal interactions at the electrophysiological level (46). This supports a paradigm where electrical signaling complements chemical communication in microbial consortia. As a result, there is an underexplored mechanistic link between physical bacterial-fungal contact and fungal electrophysiology. However, the impact of bacterial adhesion on fungal membrane potential and ion channel activity is not well elucidated. Technical barriers hinder electrophysiological exploration of fungal membranes including those relating to establishing a seal in patch-clamp studies (47). Both microelectrode array and extracellular electrochemical impedance spectroscopy measurements can be used, among other methods, to investigate this modulating capacity over varying experimental timescales.

Infection by bacteria either did not transpire at the timescale of the duration of our experiments or was not a specific aspect of the interaction due to lack of hyphal fragmentation. As a result, the bacterial species can be confirmed as an endobiont under these specific conditions and in particular as a result of the presence of the fungal EPS. Further studies would need to confirm whether the attachment structures have any functional role beyond mechanical anchoring. SEM imaging is a useful method to investigate physical attachment features of cocultures of bacteria and mycelia. In this study, the dehydration protocol used removed the exterior of the biofilm and preserved the biofilm components attached to bacteria and on the surface of the hyphae. This allowed for analysis of the retained connective structures not otherwise possible. For instance, in ESEM, hydration and humidity can be preserved. This allows for the exterior of the biofilms to be imaged, but the interior contents are completely occluded. In the dehydration series approach outlined in this paper the dehydration artifacts impacted hyphal structures. However, this was not significant in terms of interpretation of the physical characteristics and morphology of the attachment structures between hyphae and bacteria. The effects of this can be limited by reducing the number of HDMS rinses. Modification of the ethanol series percentages may also be beneficial to the hydration of the hyphae. The attachment structures anchoring the bacteria to the hyphae were preserved along with the overall morphology of the bacteria.

In the imaged samples, hydration was preserved sufficiently to view bacterial interactions with hyphal surfaces in cocultures. For investigation of overall features of the biofilm, the cocultures can be imaged at full hydration using ESEM (48). However, the density of the mycelial biofilm may prohibit imaging of components in the interior of the biofilm including bacteria and relevant surface-interfacing physical features with length scales of hundreds of nanometers. 3D microscopy methods such as lightsheet microscopy would improve these measurements, taking into account the overall colony densities. However, this kind of measurement was not possible using the preparation methods discussed above and may not resolve nanoscale details. Epoxy-based encapsulation and slicing of the resulting samples could improve understanding of 3D details in future work using SEM. Transmission electron microscopy, cryo-SEM, and similar techniques can be explored in combination with Fourier transform infrared micro-spectroscopy to add detail to augmentation of signaling modalities by these surface-interfacing and ECM enveloped ectobionts. However, the simplicity of the dehydration-graded method is a major advantage for rapid assessment of surface-interfacing features. It is expected to be especially useful in studies of contact-dependent modulation of physiology and electrophysiology of the fungal host. Dehydration methods could also be performed using ESEM and automated gradients using suitable software and hardware setups.

## Conclusion

In this paper, we outlined an approach to identifying a surface-interfacing morphological feature of ectobiont bacteria in cocultures with mycelium. Mycelium was grown in submerged liquid cultures rich in EPS. The planktonic bacteria were injected into the extracellular matrix. A graded dehydration protocol was used to selectively remove extraneous biofilm components and reveal intact bacteria and surface-interfacing features. The dehydration methods allowed for identification of specific interactions and differentiated these cultures from trivial stochastic mixing of bacteria and mycelium in liquid media.

## Data and code availability

The data sets used and/or analyzed during the current study are available from the corresponding author on reasonable request.

## Acknowledgments

The authors would like to thank David Patton for assistance with scanning electron microscopy. The research has been conducted under the framework of the FUNGATERIA (www.fungateria.eu) project, which has received funding from the European Union’s HORIZON-EIC-2021-PATHFINDER CHALLENGES programme under grant agreement no. 101071145 and 10.13039/100014013UK Research and Innovation grant no. 10048406.

## Author contributions

All authors contributed to the conceptualization and design of the study. D.B. conducted the experiments and collected the data. D.B. analyzed the data and interpreted the results. D.B. and A.A. edited and revised the manuscript. All authors reviewed and approved the final version of the manuscript.

## Declaration of interests

The authors declare no competing interests.
